# Self‐efficacy, resilience and healthy ageing among older people who have an acute hospital admission: A cross‐sectional study

**DOI:** 10.1002/nop2.1960

**Published:** 2023-08-21

**Authors:** Sarah E. Remm, Elizabeth Halcomb, Kath Peters, Deborah Hatcher, Steven A. Frost

**Affiliations:** ^1^ School of Nursing and Midwifery Western Sydney University Sydney New South Wales Australia; ^2^ School of Nursing University of Wollongong Wollongong New South Wales Australia

**Keywords:** chronic illness, healthy ageing, older people, resilience, self‐efficacy

## Abstract

**Aim:**

To examine the associations between self‐efficacy, resilience and healthy ageing among older people who have an acute hospital admission.

**Design:**

A cross‐sectional study.

**Method**s**:**

Survey and medical record data were collected from older people on discharge from hospital. The survey measured self‐efficacy with the 6‐item General Self‐Efficacy scale (GSE‐6), resilience with the Brief Resilience Scale (BRS), and healthy ageing with the Selfie Ageing Index (SAI). Medical record data included potential confounders: co‐morbidities, frailty items, previous falls and previous admission in the last 28 days. Multi‐linear regression and Spearman's rank correlation coefficient were used to examine the independent associations between self‐efficacy, resilience and healthy ageing.

**Results:**

Responses were received from 143 older people (mean age 79). After adjusting for potential confounders, co‐morbidities (*ß* = ‐0.08, *p* = 0.001) remained negatively associated with healthy ageing, while self‐efficacy (*ß* = 0.03, *p* = 0.005) and resilience (*ß* = 0.05, *p* < 0.001) remained positively associated with healthy ageing (*R* ^2^ = 0.243). Positive correlations were found between self‐efficacy (*ρ* = 0.33, *p* < 0.01), resilience (*ρ* = 0.38, *p* < 0.001) and healthy ageing. Positive correlations were also found between self‐efficacy and resilience (*ρ* = 0.38, *p* < 0.01). Those with lower self‐efficacy and resilience were more likely to report reduced activities of daily living, mobility, physical activity and mood.

**Conclusion:**

Findings indicate that while the number of co‐morbidities have negative consequences for healthy ageing among older people who are hospitalised, the promotion of self‐efficacy and resilience can potentially contribute to healthy ageing within the physical and psychological domains.

**Implications for Patient Care:**

Nurses can promote self‐efficacy, which can potentially increase resilience and help to improve self‐management of chronic conditions, functional ability in daily activities, mobility and physical activity and reduce both anxiety and depressive symptoms.

**Patient Contribution:**

Participant feedback throughout the data collection process assisted in the evaluation of study methods and data interpretation. This included processes such as assessing selected tools and clarifying the meanings of healthy ageing factors.

## 1 INTRODUCTION

In 2020, people aged 60 years and over comprised 13.5% of the world's population, and this is projected to increase to around 20% by 2050 (World Health Organization, 2020). Modernisation and globalisation have precipitated advancements in treatment options and shifts in lifestyle habits and risk factors. Consequently, more older people are living longer with chronic conditions, placing increasing pressure on healthcare services (Haugan & Eriksson, 2021). This combination of ageing populations, lifestyle risk factors and increasing morbidity highlights the complexity of health promotion in the 21st century. It is not surprising then, that modern concepts of healthy ageing are not determined by the absence of disease, but rather by maintaining the ability to carry out the functions required to have good quality of life (World Health Organization, 2020).

Healthy ageing is generally considered to be multidimensional, encompassing numerous interconnected factors from biological, psychological and social domains (Gonçalves et al., 2017; Remm et al., 2021). The intrinsic and environmental resources that older people can draw upon to maintain a level of function that facilitates quality of life are major determinants of healthy ageing (World Health Organization, 2020). However, high levels of heterogeneity among older people result in an array of different biopsychosocial challenges associated with ageing, that can impact functional abilities and quality of life (Liira et al., 2018).

Hospitalisation presents numerous risks and challenges that can threaten healthy ageing. Older people who are hospitalised are more likely to experience chronic conditions, depressive symptoms, impaired physical function and decreased social activity (Lee et al., 2022). Hospital health care is traditionally concerned with illness treatment and management, which in isolation fails to provide holistic and supportive care and recognises its potential for health promotion (Haugan & Eriksson, 2021). There are many factors, beyond the physical condition, which contribute to how older people adapt and cope with the consequences of hospital admission (Liira et al., 2018). Targeting factors that promote positive adaptive behaviours among older people who are hospitalised can improve physical, mental and social outcomes (World Health Organization, 2020).

## 2 BACKGROUND

Being resilient to the risks and challenges associated with hospitalisation is vital to the healthy ageing of older people who are hospitalised and plays an important role in recovery from illness (Haugan & Eriksson, 2021; World Health Organization, 2020). While resilience is a broad concept with varying definitions, the core notion of resilience can be thought of as engaging in positive and adaptive responses after experiencing adversity (Angevaare et al., 2020). Contemporary concepts of resilience have also considered dynamic interplays between personal and contextual factors that influence the coping processes involved in the adaptive responses of older people (Gorska et al., 2022). Similarly, concepts of healthy ageing consider the effects of intrinsic and environmental factors on being able to maintain a level of functioning that supports well‐being. This further highlights multidimensionality in older people's ability to respond to adversity (World Health Organization, 2020). Understanding factors that affect resilience in older people experiencing hospital‐associated adversity is particularly important for developing strategies targeted at promoting adaptive responses (Haugan & Eriksson, 2021).

Self‐efficacy is an intrinsic or personal resource that contributes to factors associated with healthy ageing and resilience among older people (Remm et al., 2021; Whitehall et al., 2021). It is defined as a person's belief in their capability to perform at a level required to succeed in an event that affects their life (Bandura, 1994). This belief in their capability is achieved through four processes, including cognitive, motivational, affective and selection processes, which determine the level of effort and persistence a person will commit to achieving success (Bandura, 1994). Nursing research investigating the role of self‐efficacy in health promotion has been increasing, with positive indications for health behavioural change, self‐care management and well‐being (Haugan & Eriksson, 2021). However, among those older people receiving health care, acutely hospitalised older people are more likely to have lower self‐efficacy (Whitehall et al., 2020). This highlights a need to further explore the potential implications that hospitalisation may have on the healthy ageing of older people through its impact on self‐efficacy and resilience. With projected increases in hospitalisation among older people, it is becoming increasingly important to find effective strategies that promote healthy ageing. Therefore, the current study aimed to examine associations between self‐efficacy, resilience and healthy ageing among older people who have had an acute hospital admission.

## 3 METHODS

### 3.1 Design

This paper reports data from the first phase of a sequential mixed‐method study. This phase combines data from a cross‐sectional survey with matched medical record data collected from the Health Information Exchange (HIE) database. A subsequent qualitative phase collected disparate data and so was reported separately (Authors Own).

### 3.2 Participants

Participants were recruited from the acute wards of three public hospitals in Sydney, Australia. The hospitals span two metropolitan and one rural local government area that fall within one local health district. The two metropolitan government areas serve communities with high migrant populations and areas of socioeconomic disadvantage, while the rural government area has 24.7% of the community aged over 65 years (South West Sydney PHN, 2019).

### 3.3 Sample size and power

Power was calculated using the average acute admission rates over a 3‐month period in the previous year, of people aged 65 or greater across the included hospitals. It determined a minimum of 100 participants were needed to give a power of at least 0.83 to detect a moderate correlation between healthy ageing, self‐efficacy and resilience (*ρ* 0.3 to 0.5).

### 3.4 Inclusion and exclusion criteria

People were eligible for inclusion if they were aged 65 years or older, admitted to a participating acute ward and discharged to their homes. Participants needed to have sufficient cognitive capacity and adequate English to comprehend survey questions. Cognition was assessed by the three researchers involved in data collection who were all baccalaureate‐prepared (or equivalent) Registered Nurses with specialist aged care qualifications. People were excluded if they were discharged to a residential aged care facility, supported accommodation or were considered terminally ill.

### 3.5 Data collection

Data were collected between February and May 2021. Potential participants were identified in consultation with nurse managers and invited to participate on either the day of or the day before their discharge. Time of discharge was selected to accurately measure participant's levels of self‐efficacy and resilience when being discharged home, and after experiencing hospitalisation. While ideally data were to be collected on the day of discharge, this was not always practical. Therefore, where this could not be achieved data were collected the day before a known discharge. Participants were asked to complete the survey either independently using a tablet device provided by the researcher or by verbally responding to questions with the researcher entering their responses.

### 3.6 Instruments

#### 3.6.1 Survey

The survey was developed using tools that had been previously validated and widely used in research with older people and based on relevant literature (Remm et al., 2021). The first section collected participant demographics and participants were asked to self‐rate their health on a 7‐point Likert scale (poor/bad/very bad/fair/good/very good/excellent.

The second section measured healthy ageing using the 14‐item Selfie Ageing Index (SAI) (Gonçalves et al., 2017). The SAI comprises weighted items across the biological, psychological and social domains that are rescaled to a 0–1 index score (Gonçalves et al., 2017).

The third section used the 6‐item Brief Resilience Scale (BRS) to measure resilience, that is the ability to recover from a stressor (Smith et al., 2008). The BRS has an equal distribution of positively and negatively framed items rated on a 5‐point Likert scale (strongly agree to strongly disagree). Positively framed items were scored 5 for ‘Strongly agree’ and 1 for ‘Strongly disagree’. Negatively framed items were scored in the reverse.

The final section measured self‐efficacy using the 6‐item short form of the General Self‐efficacy Scale (GSE‐6) (Romppel et al., 2013). The GSE‐6 was selected after pilot participant feedback indicated that the 10‐item version Schwarzer and Jerusalem (1995) was too long and burdensome. Items are rated on a 4‐point Likert scale (1 not at all true to 4 very true).

#### 3.6.2 Health information exchange (HIE) data

Routinely collected admitted patient (HIE) data included coded medical record data from current and previous admissions. Data collected from the survey were linked to data collected from the HIE database using participants' medical record numbers and hospital sites, which were recorded at the beginning of the survey. Extracted data included age, gender, number of co‐morbidities, previously recorded falls, number of frailty deficit items, hospital length of stay and hospital admissions within the previous 28 days.

### 3.7 Validity and reliability

All included tools were chosen for their high internal consistency. The validity of the SAI was demonstrated through its predictive value of the probability of having a doctor's visit, having depression, the number of doctor visits and the number of chronic conditions (Gonçalves et al., 2017). The Cronbach's alpha for the BRS in the current study was 0.81, whereas The Cronbach's alpha for the GSE‐6 was 0.79.

### 3.8 Data management and analysis

All data management and analysis were undertaken using R, version 1.4.1 (R Core Team, 2017). Data analysis was performed by the Doctoral candidate (SR) under the guidance of SF, an academic with extensive experience in quantitative research and statistical analysis. Demographic and health characteristics were explored using descriptive statistics. The correlation between self‐efficacy, resilience and healthy ageing was assessed using Spearman's rank correlation (Hollander et al., 2013). Stepwise multi‐linear‐regression analysis was used to estimate the independent association between self‐efficacy, resilience and healthy ageing while adjusting for age, sex, previous falls, number of co‐morbidities, number of frailty items and whether participants had an admission in the previous 28 days (Chambers & Hastie, 1992). A combined stepwise selection method was used to identify independent relationships between self‐efficacy, resilience and healthy ageing and all variables associated with SAI. The coefficient of determination (*R* ^2^) of the multi‐linear regression models is presented to estimate the variability of SAI explained by the variables included in the models. As the rate of missing data was <0.001%, no data imputation was undertaken. The chi‐square test for trend in proportions was used to assess significant trends between self‐efficacy and resilience score quartiles and dichotomised SAI items. This method was adopted to identify potentially modifiable factors of healthy ageing that may be improved through the promotion of self‐efficacy and resilience.

### 3.9 Ethical considerations

Research Ethics Committee approval was obtained from South Western Sydney Local Health District Human Research Ethics Committee (approval number 2020/ETH02406). Participants were provided with an information sheet outlining the study details, including participant rights to decline, withdraw or participate without impacting on the care received. Consent was obtained from all participants before data collection.

## 4 RESULTS

### 4.1 Participant characteristics

Of the 143 participants, 59.4% (*n* = 85) were female, and the mean age was 79 years (SD 8.4) (Table 1). Some 51.7% (*n* = 74) of participants self‐rated their health as fair, while 30.1% (*n* = 43) rated their health as good/very good/excellent. Overall, 52% (*n* = 74) of participants had one or more co‐morbidities. There were 26 participants (18.2%) with a previous fall recorded. The average length of stay in the current admission was 5 days (IQR 3–10), and 19.6% of participants (*n* = 28) had another admission within the previous 28 days. The overall mean scores were SAI 0.59 (SD = 0.15, *p =* 0.029), GSE‐6 19.3 (SD = 3.3, *p* = 0.003), and BRS 3.71 (SD = 0.84, *p* = 0.003*)*. There were no statistically significant differences between scores among the different age groups.

**TABLE 1:** Characteristics of study participants based on age group.

	65–74 (*n* = 50)	75–84 (*n* = 56)	85+ (*n* = 37)	Combined *n* = 143	*p* value
SAI score (0–1), mean (SD)	0.60 (0.16)	0.59 (0.15)	0.59 (0.15)	0.59 (0.15)	0.029
GSE score (6–24), mean (SD)	19.3 (3.6)	19.7 (3.0)	18.8 (3.4)	19.3 (3.3)	0.003
BRS score (1–5), mean (SD)	3.51 (0.9)	3.82 (0.9)	3.82 (0.7)	3.71 (0.8)	0.003
Women, *n* (%)	26 (52.0)	34 (60.7)	25 (67.6)	85 (59.4)	0.330
Self‐rated health, *n* (%)					0.310
Fair	26 (52.0)	24 (42.9)	24 (64.9)	74 (51.7)	
Good/very good/excellent	15 (50.0)	21 (37.5)	7 (18.9)	43 (30.1)	
Poor/bad/very bad	9 (18.0)	11 (19.6)	6 (16.2)	26 (18.2)	
Number of co‐morbidities, *n* (%)					0.820
0 co‐morbidities	25 (50.0)	26 (46.4)	18 (48.6)	69 (48.3)	
1 co‐morbidity	18 (36.0)	20 (35.7)	13 (35.1)	51 (35.7)	
2–5 co‐morbidities	7 (28.0)	10 (17.9)	6 (16.2)	23 (16.1)	
Previous falls, *n* (%)	5 (10.0)	10 (17.9)	11 (29.7)	26 (18.2)	0.062
Hospital length of stay (days), median (IQR)	7.1 (3–8)	9.2 (3–9)	9.2 (3–15)	8.5 (3–10)	0.560
Admission in last 28 days, *n* (%)	9 (18.0)	14 (25.0)	5 (13.5)	28 (19.6)	0.310

*Note*: Continuous variables were compared using a Kruskal–Wallis and categorical variables using Pearson's Chi‐squared test.

### 4.2 Association between healthy ageing, self‐efficacy and resilience

The Spearman's rank correlation showed a positive correlation between self‐efficacy and healthy ageing (*ρ* = 0.33, *p* < 0.001), and between resilience and healthy ageing (*ρ* = 0.38, *p* < 0.001). A positive correlation was also found between general self‐efficacy and resilience (*ρ =* 0.38, *p* < 0.01*)*.

### 4.3 Trends among self‐efficacy, resilience and specific healthy ageing items

Tables 2 and 3 present the scores for healthy ageing items according to self‐efficacy and resilience score quartiles. Those who had lower self‐efficacy were significantly more likely to report deficits in activities of daily living (ADLs) (*p* = 0.03), undertaking moderate physical activity once or less than once a week (*p* = 0.01), feeling depressed (*p* = 0.01), feeling nervous (*p* = 0.01) and lacking energy (*p* = 0.1). Similarly, those with lower resilience were significantly more likely to report deficits in ADLs (*p* = 0.07), mobility difficulties indoors (*p* = 0.01), undertaking moderate physical activity once or less than once a week (*p* = 0.03), feeling depressed (*p* < 0.001), feeling nervous (*p* < 0.001) and lacking energy (*p* = 0.02).

**TABLE 2:** SAI item scores in quartiles of self‐efficacy scores.

	General self‐efficacy score quartiles				*p* value
	8–18 (*n* = 40)	18–20 (*n* = 34)	20–23 (*n* = 37)	23–24 (*n* = 31)	
Oriented to year/month/day of month/day of the week, *n* (%)					0.66*
Minimum of 3 out of 4 correct	37 (92.5)	30 (88.2)	35 (94.6)	29 (93.5)	
BMI (mg/m^2^), mean (SD)	29.8 (6.5)	29.0 (9.1)	28.4 (9.7)	29.4 (7.9)	0.60
Smoking status, mean (%)					0.31
Current	5 (12.5)	0 (0)	3 (8.1)	2 (6.5)	
Former	14 (35.0)	20 (58.8)	16 (43.2)	15 (48.4)	
Never	21 (52.5)	14 (41.2)	18 (48.6)	14 (45.2)	
Number of deficits in basic ADLs, *n* (%)					0.03*
0–1 deficits	27 (67.5)	27 (79.4)	31 (83.8)	27 (87.1)	
2–5 deficits	13 (32.5)	7 (20.6)	6 (16.2)	4 (12.9)	
Difficulties with mobility indoors, *n* (%)	13 (32.5)	5 (14.7)	7 (18.9)	6 (19.4)	0.21*
Vigorous physical activity less than once a week, *n* (%)	32 (80.0)	28 (82.4)	27 (73.0)	21 (67.7)	0.16*
Moderate physical activity only once or less than once a week, *n* (%)	20 (50.0)	18 (52.9)	7 (18.9)	9 (29.0)	0.01*
Felt depressed in the last month, *n* (%)	26 (65.0)	13 (38.2)	17 (45.9)	10 (32.3)	0.01*
Felt nervous in the last month, *n* (%)	20 (50.0)	10 (29.4)	14 (37.8)	7 (22.6)	0.04*
Lack of energy in the last month, *n* (%)	31 (77.5)	25 (73.5)	22 (59.5)	20 (64.5)	0.10*
Has someone to confide in, *n* (%)	33 (82.5)	28 (82.4)	31 (83.8)	27 (87.1)	0.60*
Marital status, *n* (%)					0.90
Married/de‐facto	22 (55.0)	20 (58.8)	19 (51.4)	14 (45.2)	
Widowed	10 (25.0)	10 (29.4)	12 (32.4)	11 (35.5)	
Divorced	6 (15.0)	3 (8.8)	5 (13.5)	3 (9.7)	
Single	2 (5.0)	1 (2.9)	1 (2.7)	3 (9.7)	
Main type of work as non‐manual, *n* (%)	21 (52.5)	14 (41.2)	14 (37.8)	13 (41.9)	0.30*
Education in years, mean (SD)	11.2 (2.9)	11.6 (3.5)	11.2 (3.4)	12.3 (2.5)	0.32

*Note*: *p* values are from Pearson's chi‐squared test or Kruskal–Wallis, for categorical or continuous variables, respectively. Except for trend tests where indicated (*).

**TABLE 3:** SAI item scores in quartiles of BRS scores.

	Brief resilience sore range in quartiles				
	1.17–3.17 (*n* = 43)	3.17–3.83 (*n* = 30)	3.83–4.5 (*n* = 36)	4.5–5.0 (*n* = 34)	*p* value
Oriented to year/month/day of month/day of the week, *n* (%)					
Minimum of 3 out of 4 correct	40 (93.0)	26 (86.6)	33 (91.7)	32 (94.1)	0.77*
BMI (mg/m^2^), mean (SD)	30.1 (9.2)	29.5 (7.2)	27.6 (6.7)	29.5 (9.5)	0.57
Smoking status, mean (SD)					0.57
Current	4 (9.3)	3 (10.0)	2 (5.6)	1 (2.9)	
Former	19 (44.2)	12 (40.0)	21 (58.3)	14 (41.2)	
Never	20 (46.5)	15 (50.0)	13 (36.1)	19 (55.9)	
Number of deficits in basic ADLs, *n* (%)					0.07*
0–1 deficits	32 (74.4)	20 (66.7)	30 (83.3)	30 (88.2)	
2–5 deficits	11 (25.6)	10 (33.3)	6 (16.7)	4 (11.8)	
Difficulties with mobility indoors, *n* (%)	14 (32.6)	9 (30.0)	6 (16.7)	3 (8.8)	0.01*
Vigorous physical activity less than once a week, *n* (%)	33 (76.7)	23 (76.7)	28 (77.8)	25 (73.5)	0.80*
Moderate physical activity only once or less than once a week, *n* (%)	22 (51.2)	12 (40.0)	11 (30.6)	10 (29.4)	0.03*
Felt depressed in the last month, *n* (%)	27 (62.8)	18 (60.0)	14 (38.9)	8 (23.5)	<0.001*
Felt nervous in the last month, *n* (%)	27 (62.8)	15 (50.0)	5 (13.9)	4 (11.8)	<0.001*
Lack of energy in the last month, *n* (%)	35 (81.4)	22 (73.3)	22 (61.1)	20 (58.8)	0.02*
Has someone to confide in, *n* (%)	35 (81.4)	26 (86.7)	30 (83.3)	29 (85.3)	0.72*
Marital status, *n* (%)					0.35
Married/de‐facto	26 (60.5)	15 (50.0)	18 (50.0)	16 (47.1)	
Widowed	9 (20.9)	9 (30.0)	10 (27.8)	16 (47.1)	
Divorced	5 (11.6)	5 (16.7)	5 (13.9)	2 (5.8)	
Single	3 (7.0)	1 (3.3)	3 (8.3)	0 (0.0)	
Main type of work as non‐manual, *n* (%)	16 (37.2)	14 (46.7)	17 (47.2)	15 (44.1)	0.50*
Education in years, mean (SD)	10.8 (3.6)	11.1 (3.4)	12.2 (2.4)	12 (2.8)	0.13

*Note*: *p* values are from Pearson's chi‐squared test or Kruskal–Wallis, for categorical or continuous variables, respectively, except for trend tests where indicated (*).

### 4.4 Independent association between self‐efficacy, resilience and healthy ageing

After adjusting for age, sex, previously recorded falls, frailty items, number of co‐morbidities and having an admission in the last 28 days, the most significant contributor to healthy ageing was the number of co‐morbidities (*ß* = ‐0.08; CI 95% ‐0.12, −0.03, *p* = 0.001), followed by resilience (*ß* = 0.05; CI 95% 0.01, 0.06, *p* < 0.001) and self‐efficacy (*ß* = 0.03; CI 95% 0.01, 0.06, *p* = 0.005) (Table 4). The coefficient of determination (*R* ^2^) for this final model was 0.243.

**TABLE 4:** Association between self‐efficacy, resilience and healthy ageing.

	Unit(s) of analysis	Univariate model coefficient (95% CI)	Multivariate model coefficient (95% CI)	Final model coefficient (95% CI)	Adj. *p* value
GSE	1‐SD increase	0.05 (0.03 to 0.07)	0.03 (0.01 to 0.05)	0.03 (0.01 to 0.06)	0.005
BRS	1‐SD increase	0.06 (0.03 to 0.08)	0.05 (0.02 to 0.07)	0.05 (0.02 to 0.07)	<0.001
Males	Versus females	0.02 (−0.03 to 0.07)	0.00 (−0.04 to 0.05)		
Age	Each 5‐year increase	−0.01 (−0.02 to 0.01)	−0.01 (−0.02 to 0.01)		
Previous falls	Versus no falls	−0.02 (−0.09 to 0.04)	−0.01 (−0.07 to 0.06)		
Number of frailty items	Each 2 increase	−0.04 (−0.07 to −0.01)	−0.01 (−0.0 to 0.03)		
Number of co‐morbidities	Each 2 increase	−0.06 (−0.11 to −0.01)	−0.06 (−0.13 to 0.00)	−0.08 (−0.12 to −0.03)	0.001
Admissions the in last 28 days	Versus no admission in 28 days	−0.04 (−0.10 to 0.03)	0.02 (−0.07 to 0.04)		
*R* ^2^		NA	0.257	0.243	

## 5 DISCUSSION

This study investigated the associations between self‐efficacy, resilience and healthy ageing among older people who have had an acute hospital admission. The results confirm that co‐morbidities negatively contribute to healthy ageing among older people who have an acute admission, while greater self‐efficacy and resilience positively contribute to healthy ageing. Self‐efficacy was also positively associated with resilience, supporting previous literature identifying self‐efficacy as a key determinant of resilience (Whitehall et al., 2021). This suggests that increasing self‐efficacy may have the benefit of not only promoting healthy ageing but also enhancing resilience among older people in acute hospital settings. Furthermore, those reporting lower self‐efficacy and resilience were more likely to have difficulties with daily activities, limitations in mobility and physical activity and mood disturbances.

Hospitalisation is associated with numerous factors that can have negative consequences for the biopsychosocial domains of healthy ageing (Lee et al., 2022). In the current study, the number of co‐morbidities was identified as having a significant negative contribution towards healthy ageing. Multi‐morbidity is associated with numerous poor health outcomes such as functional decline, frailty, poor mental health and increased healthcare utilisation, which contribute to a lower quality of life (Kuzuya, 2019). Effective management of chronic conditions is important for healthy ageing and is influenced by person‐oriented resources such as self‐efficacy and resilience (Haugan & Eriksson, 2021). Both self‐efficacy and resilience have previously been found to moderate the negative consequences of chronic conditions and help to improve self‐management (Manning et al., 2016; Schüz et al., 2012). This suggests that an awareness of older people's level of self‐efficacy and resilience may help to identify those who may benefit from intervention. Potential benefits of enhancing self‐efficacy and resilience among hospitalised older people with multi‐morbidity may include improving health outcomes, leading to greater quality of life.

Functional decline is common and often prolonged for older people who have been hospitalised (Dharmarajan et al., 2020). This can result in negative consequences for coping and health behaviours that promote adaptive responses needed to reduce the risk of poor health‐related outcomes (Haugan & Eriksson, 2021). This study identified lower self‐efficacy and resilience to be associated with greater deficits in ADLs, mobility and physical activity, indicating a potential benefit to functional capacity through improved self‐efficacy and resilience. Similar findings support self‐efficacy's ability to mediate factors such as pain and negative ageing‐related beliefs, which can negatively impact ADLs and physical activity (Schulz et al., 2015; Yeom, 2014). Improved functional capacity has also previously been found to be associated with higher levels of resilience among older people who were hospitalised (Bezerra Leão et al., 2018). However, not all literature supports this association. A recent review of factors associated with resilience among older people found the relationship between resilience and ADLs to be non‐significant (Gorska et al., 2022). The same review found that higher scores in personal factors such as health‐promoting lifestyle and self‐efficacy had strong positive associations with resilience. When considering self‐efficacy's positive mediating relationship with health‐promoting lifestyle behaviours (Yeom, 2014), this further suggests that enhancing levels of self‐efficacy may improve functional independence by mediating health behaviours and resilience.

Many older people who are hospitalised experience depressive symptoms and anxiety, which can significantly reduce well‐being (Lee et al., 2022). Depression can limit intrinsic resources, such as resilience, and result in serious health consequences for older people, leading to reduced quality of life and physical functioning (Haugan & Eriksson, 2021). Depressive symptoms and anxiety have also been reported to be associated with lower levels of resilience and self‐efficacy among older people both in community and residential aged‐care settings (Cybulski et al., 2017; Gorska et al., 2022). Considering the previously highlighted risk of reduced self‐efficacy among acutely hospitalised older people (Whitehall et al., 2020), this suggests that there may be an increased vulnerability to depressive symptoms and anxieties related to their hospital experience. However, as highlighted in previous literature, interventions aimed at increasing self‐efficacy and resilience can help mediate negative relationships between health problems and depressive symptoms (Lim et al., 2015; O'Shea et al., 2016). This further supports the potential benefits of implementing strategies aimed at improving self‐efficacy and resilience as a way of promoting both physical function and mental well‐being.

### 5.1 Strengths and limitations

The results of this study needed to be considered in the context of some potential strengths and limitations. The collection of objective data from medical records helped to strengthen this study, as it allowed for the adjustment of several potential confounders within the multi‐regression model. Another strength was that generalisability was increased by including participants from multiple hospitals. These facilities served diverse urban communities, with significant migrant and low socioeconomic groups, and rural areas. Despite the diversity in local communities, consideration of these characteristics is important before generalising these findings to other settings.

There were some limitations of the study. Firstly, the cross‐sectional design, where causality cannot be assumed, limited findings. A further limitation was the English language requirement, which excluded some potential participants from non‐English speaking backgrounds. Furthermore, due to the geographical distance between hospitals and difficulties accessing acute wards during COVID‐19 restrictions, some potential participants were missed. While this limited the sample size, the number of participants still exceeded the minimum required to detect a moderate degree of correlation determined through power calculation. Finally, the tools used in this study were chosen due to their widespread use and appropriateness for the general population of older people. Future research may consider using disease‐specific tools with individual disease groups to tease out the nuances between specific chronic conditions and self‐efficacy.

### 5.2 Clinical implications

The findings of this study support the need for health promotion activities within hospitals to go beyond traditionally limited interventions focused on disease treatment and management and include strategies aimed at building resilience and self‐efficacy. Nurses can identify older people at risk of diminished healthy ageing factors, and incorporate strategies to support self‐efficacy and resilience into nursing care plans. Strategies that enhance self‐efficacy may be particularly useful in older people with multi‐morbidity or functional problems with mobility, ADLs and physical activity, and those experiencing anxiety or depression. For example, incorporating verbal persuasion and mastery techniques to encourage persistent effort in carrying out ADLs can help improve adaptive coping and reduce the risk of functional decline and disability. Furthermore, recognising the interconnectedness among the biological, psychological and social domains can help nurses tailor their interventions to meet the healthy ageing needs of older people.

## 6 CONCLUSION

Findings from this study highlight the negative impact of multi‐morbidity on healthy ageing among older people who are acutely hospitalised. It also highlights several potential benefits that the promotion of self‐efficacy and resilience can contribute to the physical and psychological domains of healthy ageing among older people who experience hospitalisation. Health promotion strategies that improve self‐efficacy can also potentially increase resilience and help to improve functional abilities and reduce anxiety and depressive symptoms. Enhancing self‐efficacy may also serve to buffer the negative impact of multimorbidity on the healthy ageing of acutely hospitalised older people through potential improvements to self‐management of chronic conditions. Future research that explores the personal perspectives and experiences of acutely hospitalised older people would provide further insight into adaptive responses and inform strategies aimed at promoting healthy ageing.

## AUTHOR CONTRIBUTIONS

Sarah E. Remm: Conceptualisation, Methodology, Data collection, Analysis, Writing—original draft. Kath Peters, Elizabeth Halcomb and Deborah Hatcher: Review and editing, Supervision. Steven A. Frost: Methodology, Analysis, Review and editing, Supervision.

## FUNDING INFORMATION

This study was **s**upported by the Hazlett Family Foundation Postgraduate Research Scholarship.

## CONFLICT OF INTEREST STATEMENT

The author(s) declared no potential conflicts of interest with respect to the research, authorship and/or publication of this article.

## ETHICS STATEMENT

Research Ethics Committee approval was obtained from the South Western Sydney Local Health District Human Research Ethics Committee (approval number 2020/ETH02406).

## HANDLING OF STATISTICAL DATA STATEMENT

The authors have ensured that our submission conforms as applicable to the Journal's statistical guidelines. Author Steven Frost has extensive experience in statistical analysis and has consulted with author Sarah Remm in all statistical analyses and reporting of data within this manuscript. The authors affirm that the methods used in the data analyses, which include multi‐linear regression and Spearman's rank correlation, are suitably applied to the study's data within the study design and context, and that statistical findings have been implemented and interpreted correctly.

## Data Availability

The data that support the findings of this study are available from the corresponding author upon request. The data are not publicly available due to privacy or ethical restrictions.
